# A universal long-term flu vaccine may not prevent severe epidemics

**DOI:** 10.1186/1756-0500-3-92

**Published:** 2010-04-05

**Authors:** Raffaele Vardavas, Romulus Breban, Sally Blower

**Affiliations:** 1 RAND Corporation, Santa Monica, California, USA; 2 Institut Pasteur, Paris, France; 3 Semel Institute for Neuroscience and Human Behavior, University of California Los Angeles, Los Angeles, California, USA

## Abstract

**Background:**

Recently, the promise of a new universal long-term flu vaccine has become more tangible than ever before. Such a vaccine would protect against very many seasonal and pandemic flu strains for many years, making annual vaccination unnecessary. However, due to complacency behavior, it remains unclear whether the introduction of such vaccines would maintain high and stable levels of vaccination coverage year after year.

**Findings:**

To predict the impact of universal long-term flu vaccines on influenza epidemics we developed a mathematical model that linked human cognition and memory with the transmission dynamics of influenza. Our modeling shows that universal vaccines that provide short-term protection are likely to result in small frequent epidemics, whereas universal vaccines that provide long-term protection are likely to result in severe infrequent epidemics.

**Conclusions:**

Influenza vaccines that provide short-term protection maintain risk awareness regarding influenza in the population and result in stable vaccination coverage. Vaccines that provide long-term protection could lead to substantial drops in vaccination coverage and should therefore include an annual epidemic risk awareness programs in order to minimize the risk of severe epidemics.

## Discussion

### Influenza vaccination behavior and universal flu vaccines

Influenza is the lead cause of death from a vaccine-preventable disease in the United States (US). Although about 80% of the US population is specifically recommended for annual influenza vaccination, less than 40% of the population usually gets vaccinated [1]. Despite the rising vaccination rates in recent years, these still fall short of *Healthy People 2010 *objectives [2,3]. Hopes are that the introduction of a new vaccine offering long-term protection over many years would lead to a significantly increase in the vaccination coverage. Recently, the possibility of developing such universal flu vaccines has become more tangible than ever before [4,5]. In early 2008, Acambis of Cambridge, Massachusetts (now Sanofi Pasteur) reported positive results for a phase 1 clinical trial of a universal vaccine [6]. Independently that same year, a group at Oxford, England, led by Dr. Gilbert started a phase 1 clinical trial of another universal flu vaccine that would provide protection for at least 5-10 years after which a booster will be required [7]. More recently, lab-made proteins have been identified which would allow the vaccine to neutralize a broad range of influenza strains, including the 1918 pandemic strain [8]. Such universal vaccines would provide for the possibility of building up long-lasting herd immunity in the population and prevent epidemics. However, their success will still depend upon the vaccination coverage that can be achieved. Long-lasting herd immunity may lead to complacency behavior and it remains unclear whether the introduction of such vaccines would maintain high and stable levels of vaccination coverage year after year.

### The "free rider problem"

Currently, annual vaccination in the US is provided on a voluntary basis. When vaccination is voluntary some individuals may avoid annual vaccination. In some years these individuals may be protected from infection as a result of a high level of herd immunity (i.e., they act as "free riders"). When the levels of herd immunity are kept high over many years (i.e., vaccination coverage is high), epidemics will stay small. This could increasingly lead to individuals deciding that vaccination is no longer necessary and adopt a free rider strategy. If the number of free riders increases by large amounts over a short period of time (1 or 2 years), the vaccination coverage will fall to a low level and hence a severe epidemic will occur [9,10]; this is known as a "free rider problem" [11]. In the years following a severe epidemic, many individuals in the population will again be motivated to vaccinate and therefore the level of herd immunity will begin to increase. However, once herd immunity reaches a high level the free rider problem can reoccur.

### Modeling vaccination interventions

Our work [9,10], based on modeling the impact of current influenza vaccines, has shown that it is unlikely (due to the existence of free riders) that annual voluntary vaccination will prevent severe epidemics. To predict the impact of a variety of vaccination interventions we developed a mathematical model that linked human cognition and memory with the transmission dynamics of influenza. In the model, individual-level behavior (based upon cognition and memory) drives the epidemiology, which in turn drives individual-level behavior. We modeled individuals making annual vaccination decisions (i.e., to vaccinate or not) based on remembering the outcome of their previous vaccination decisions (i.e., their "infection history" over a specified number of years; i.e., ~3-4 years) [12]. Under these conditions, the free rider problem occurs which leads to recurrent severe influenza epidemics. However, we also found that severe epidemics could be avoided if a vaccination incentive is offered; specifically, if free shots (for a given number (*y*) of years) are offered to individuals who agree to be vaccinated for the next *y-1 *years [9,10]. A universal long-lasting flu vaccine that offers protection for multiple years is analogous to this type of vaccination incentive. Here, we apply and adapt our previously developed theory to understanding the potential public health impact of universal influenza vaccines. The model that we use is described in the appendix.

### Universal vaccines versus the free rider problem

**Figure 1:**
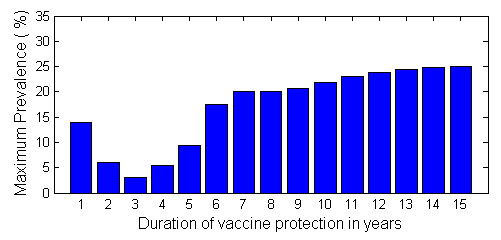
**Modeling results**. Maximum of the prevalence time series versus the duration of vaccine protection in a population that gets vaccinated on a voluntary basis with a universal vaccine.

Our modeling shows that universal vaccines that provide short-term protection (i.e., ~3-4 years) are likely to result in small frequent epidemics, whereas universal vaccines that provide long-term protection (i.e., ~8-12 years) are likely to result in severe infrequent epidemics (see Figure 1). This difference in epidemiology is the result of human cognition and memory altering the vaccination behavior that then creates the "free rider" problem. Epidemics occur when universal vaccines provide only short-term protection as some vaccinated individuals choose not to vaccinate when their vaccine protection has waned. These individuals choose to change their behavior because, during the years they are protected by vaccination, they gradually become complacent as they see that epidemics are small. Therefore some conclude vaccination is unnecessary and choose to become free riders. The number of free riders remains small because, since epidemics are frequent, many continue to believe that vaccination is necessary. Consequently, a high vaccination coverage is achieved each year and epidemics remain small. These small epidemics occur frequently, because individuals can choose to change their vaccination behavior every few years when the protective effect of the vaccine has waned. In our model, memory and complacency also determine the free rider problem for universal long-lasting vaccines. However, we found that when protection is long-term (i.e., ~8-12 years) infrequent severe epidemics will occur. In this case, the tendency of individuals to become free riders builds-up in the years between the infrequent epidemics and increasing number of individuals become complacent. Therefore, vaccination coverage falls and finally a severe epidemic occurs. Epidemics occur infrequently because individuals only have the opportunity to make decisions about vaccination every ~8-12 years. In the year after a severe epidemic a high proportion of the population will choose to vaccinate and will then need to make vaccination decisions ~8-12 years later. This synchronization of vaccination cycles exacerbates the severity of the infrequent epidemics.

## Summary and Discussion

We have constructed a model of influenza transmission dynamics coupled to human cognition and memory to address the potential problem that individuals may increasingly act as free riders and become complacent towards influenza vaccination once a universal flu vaccine has been made available. Our model shows that this behavioral mechanism may lead to infrequent but severe influenza epidemics when the vaccine provides protection for many years (~8-12 years) even without a pandemic strain. If instead the duration of protection compares to the duration of influenza vaccination memories (in our model ~3-4 years), then the introduction of a universal vaccine would lead to more stable yearly prevalence pattern without severe epidemics.

We note that universal influenza vaccines may turn out to be imperfect. For example, they may not protect from all influenza subtypes. They could also induce influenza strains to mutate in unexpected ways and thus demanding frequent updates. It is also feared that universal vaccines will not be very immunogenic, allowing for increased protection but not to the extent of preventing influenza epidemics. Nevertheless, free riders will occur even with imperfect vaccines as long as the vaccination of some individuals benefits the others (i.e., provides "herd benefits"). Furthermore, the duration of the benefit of vaccination and the vaccination memories of individuals are critical time-scale parameters that govern the dynamics of the vaccination coverage.

In conclusion, based on our modeling, we recommend that public health intervention using universal vaccines that offer long-term protection (i.e., ~8-12 years) should include an epidemic risk awareness program in order to reduce complacency with vaccination and minimize the risk of severe influenza epidemics. In contrast, public health intervention using universal vaccines that offer short-term protection (i.e., ~3-4 years) may not need this precaution. Current influenza awareness programs do stress the importance of vaccination as well as personal hygiene practices to help prevent transmission [13-16]. However, in general, the emphasis is placed on awareness of pandemics due to emerging strains. Here we argue both for the cases of emerging and non-emerging strains that, especially when using a universal vaccine offering long-term protection, more attention should be given to the fact that individuals may become complacent with influenza vaccination and act as free riders.

## Appendix: Model description

We consider a population consisting of *N *individuals acting in their own self-interest. Each individual makes personal decisions as to whether or not get vaccinated against influenza. The collective of these decisions drives influenza epidemiology that, in turn, affects future individual-level decisions. The model proceeds iteratively as follows.

At the beginning of each influenza season, every individual decides whether or not to get vaccinated against the flu depending on their immune status and their experience with flu vaccination. We assume that the vaccine offers complete protection for a certain number of years. If individuals have been vaccinated in previous years and the vaccine did not wane yet, then they are immune and will not get vaccinated. Otherwise, they will get vaccinated with a certain probability depending on their cumulative experience with flu vaccination. An epidemic occurs every influenza season, depending on the achieved vaccination coverage *p*, as described by the Susceptible-Infected-Recovered model. Thus, if the vaccination coverage exceeds a critical value, (i.e., "critical coverage") then the number of infected is zero and epidemics are prevented. Otherwise, epidemics occur, the fraction of infected *q(p) *decreasing approximately linearly with the vaccination coverage *p*. We assume that every susceptible risks infection with probability *q(p)*.

At the end of the influenza season, individuals evaluate their new experiences. We assume that individuals evaluate experience as positive if (i) they did not get vaccinated, yet avoided infection (i.e., they were free riders) or (ii) an epidemic took place while they were immunized by vaccination, and negative if (i) they vaccinated and no epidemic took place or (ii) they did not get vaccinated and got infected. Then, the pro-vaccination experience of every individual is cumulated by adding her/his number of positive experiences that occurred in the last influenza season to her/his previously gathered pro-vaccination experience now discounted by a "memory-loss" factor between 0 and 1. The probability of getting vaccinated for the next influenza season (if the vaccine wanes), is given by the cumulative pro-vaccination experience normalized by its maximum possible value. Then, the whole process repeats in the next influenza season.

The model described here is similar to the "basic model" with the second public health incentive where individuals who get vaccinated are offered free vaccinations in subsequent years; see Breban et al. [9] and Vardavas et al. [10].

## Competing interests

The authors declare that they have no competing interests.

## Authors' contributions

RV, RB and SB developed the concept and study, analyzed and interpreted the results and drafted the manuscript. RV and RB implemented and ran the model. All authors have read and approved the final manuscript.
